# Evaluation of two sets of immunohistochemical and Western blot confirmatory methods in the detection of typical and atypical BSE cases

**DOI:** 10.1186/1756-0500-4-376

**Published:** 2011-09-29

**Authors:** Chiara Porcario, S Mark Hall, Francesca Martucci, Cristiano Corona, Barbara Iulini, Alice Z Perazzini, Pierluigi Acutis, Amir N Hamir, Christina M Loiacono, Justin J Greenlee, Jürgen A Richt, Maria Caramelli, Cristina Casalone

**Affiliations:** 1 CEA, Istituto Zooprofilattico Sperimentale del Piemonte, Liguria e Valle d'Aosta, Via Bologna 148, 10154, Turin, Italy; 2 United States Department of Agriculture, Animal and Plant Health Inspection Service (APHIS), National Veterinary Services Laboratories (NVSL), Pathobiology Laboratory, 1920 Dayton Ave, Ames, IA, 50010, USA; 3 Virus and Prion Research Unit, National Animal Disease Center, United States Department of Agriculture, Agricultural Research Service (ARS), 1920 Dayton Ave, Ames, IA, 50010, USA; 4 M. D. Anderson Cancer Center, Department of Veterinary Medicine and Surgery - Unit 63, 1515 Holcombe Boulevard, Room TB.4055C, Houston, TX 77030-4009, USA; 5 K224B Mosier Hall College of Veterinary Medicine, Kansas State University, Manhattan, KS 66506-5601, USA

## Abstract

**Background:**

Three distinct forms of bovine spongiform encephalopathy (BSE), defined as classical (C-), low (L-) or high (H-) type, have been detected through ongoing active and passive surveillance systems for the disease.

The aim of the present study was to compare the ability of two sets of immunohistochemical (IHC) and Western blot (WB) BSE confirmatory protocols to detect C- and atypical (L- and H-type) BSE forms.

Obex samples from cases of United States and Italian C-type BSE, a U.S. H-type and an Italian L-type BSE case were tested in parallel using the two IHC sets and WB methods.

**Results:**

The two IHC techniques proved equivalent in identifying and differentiating between C-type, L-type and H-type BSE. The IHC protocols appeared consistent in the identification of PrP^Sc ^distribution and deposition patterns in relation to the BSE type examined. Both IHC methods evidenced three distinct PrP^Sc ^phenotypes for each type of BSE: prevailing granular and linear tracts pattern in the C-type; intraglial and intraneuronal deposits in the H-type; plaques in the L-type.

Also, the two techniques gave comparable results for PrP^Sc ^staining intensity on the C- and L-type BSE samples, whereas a higher amount of intraglial and intraneuronal PrP^Sc ^deposition on the H-type BSE case was revealed by the method based on a stronger demasking step.

Both WB methods were consistent in identifying classical and atypical BSE forms and in differentiating the specific PrP^Sc ^molecular weight and glycoform ratios of each form.

**Conclusions:**

The study showed that the IHC and WB BSE confirmatory methods were equally able to recognize C-, L- and H-type BSE forms and to discriminate between their different immunohistochemical and molecular phenotypes. Of note is that for the first time one of the two sets of BSE confirmatory protocols proved effective in identifying the L-type BSE form. This finding helps to validate the suitability of the BSE confirmatory tests for BSE surveillance currently in place.

## Background

Transmissible spongiform encephalopathies (TSEs), or prion diseases, are fatal neurological disorders in both animals and humans. TSEs include bovine spongiform encephalopathy (BSE) in cattle, scrapie in sheep and goats, transmissible mink encephalopathy (TME) in mink and Creutzfeldt-Jakob disease (CJD) in humans. The agent of TSEs has been ascribed to an infectious protease-resistant isoform of the normal host encoded cellular prion protein (PrP^C^), termed pathological prion protein (PrP^Sc^) [1].

BSE was first detected in the United Kingdom (UK) in 1986 [2], where the disease grew to epidemic proportions in the cattle population, with approximately 184,600 cases diagnosed [3]. An estimated 3 million cattle may have been infected. Worldwide, about 7,900 BSE cases have been reported outside the UK, including Canada, Japan and the United States (U.S.) [4].

Passive and active surveillance programs were developed to monitor cattle populations for the presence of BSE. Under passive surveillance, cattle showing clinical symptoms consistent with BSE must be tested for the disease. Active surveillance programs require BSE testing by rapid screening tests at the abattoir of apparently healthy cattle of a certain age and of fallen/dead stock.

A major public health concern due to its transmissibility to humans, BSE is thought to be the cause of the fatal human prion disorder variant Creutzfeldt-Jakob disease (vCJD) [5,6]. Besides transmission among cattle and to humans, BSE can cross the species barrier to other species such as small ruminants [7,8], exotic ungulates [9], and felids [10].

The origin of BSE remains an enigma: one hypothesis is that the disease was acquired by the cattle population through the consumption of meat and bone meal (MBM) derived from scrapie-affected sheep and goats or from BSE-infected cattle [11,12]. The efficiency of MBM feeding bans, which subsequently led to a worldwide decline in BSE among the cattle population, supports a food related spread of the disease.

Another hypothesis is that BSE may have arisen as a sporadic [13,14] or genetic [15,16] disease of cattle, which was then amplified by food-borne recycling of MBM containing central nervous system (CNS) tissues from BSE-infected cattle.

It was once thought that BSE was the result of a single prion strain. The disease presented with an identical clinical and pathological phenotype in cattle [17], with homogenous and consistent PrP^Sc ^molecular features and deposition patterns in the brainstem, independently of the geographical origin of the BSE isolates detected [18-21] or breed or route of inoculation in cases of experimental transmission [22]. In transmission studies using mouse models, the BSE strain was characterized by similar incubation time, biochemical features, lesion profile and PrP^Sc ^distribution regardless of origin [23,24]. But as diagnostic techniques were further refined and the BSE epidemic continued to decline, "atypical" cases of the disease, mainly in older cattle, began to be reported from around the world. The new variants were classified as low (L-) or high (H-) type based on differences in the molecular size of the PrP^Sc ^unglycosylated protein band visualized on Western blot (WB) assays as compared to classical BSE (C-type BSE) [21].

Research into more detailed characterization of PrP^Sc ^deposition throughout the brain of BSE-affected cattle uncovered the first example of atypical L-type BSE in Italy in 2004 [25]. Two older animals were found to be affected by a variant form of BSE, classified as L-type, based on WB analysis demonstrating faster electrophoretic mobility of PrP^Sc ^unglycosylated moiety. WB analysis also revealed a dominant monoglycosylated PrP^Sc ^glycoform profile for these two atypical cases. Brain samples from three additional Italian BSE-affected cattle were noted to display the same molecular features and were subsequently confirmed as atypical L-type cases.

Immunohistochemical analysis of brain sections revealed differences in the distribution and features of PrP^Sc ^immunoreactivity between the C-type BSE and the Italian L-type BSE cases. Specifically, in three Italian L-type BSE cases out of the five described so far, PrP^Sc ^was found to be more abundant in the forebrain than in the brainstem where PrP^Sc ^deposition was predominant in the C-type BSE cases. Unexpectedly, PrP^Sc ^deposition in three out of these five Italian L-type BSE cases occurred in an unusual pattern referred to as amyloid plaques, which is seldom reported in the literature for C-type BSE [26]. Availability of only the brainstem area for the fourth Italian L-type BSE case precluded full characterization for PrP^Sc ^plaques. At the time of this writing, the fifth L-type BSE case was undergoing characterization studies for PrP^Sc ^plaque deposits.

On the basis of the unique neuropathological feature of PrP^Sc ^amyloid plaques, the new L-type variant was named bovine amyloidotic spongiform encephalopathy (BASE).

The discovery of the Italian case occurred concomitantly with the detection of a second atypical BSE variant, the H-type, reported in France in 2004 [27]. H-type cases were characterized by a higher molecular weight of the unglycosylated fraction of PrP^Sc ^than C-type BSE and by strong labelling with P4 [28] and 12B2 [29] monoclonal antibodies. Both antibodies are directed to an N-terminal epitope that is still present after proteinase K (PK) cleavage of H-type BSE and also scrapie, but not of C-type or L-type BSE-derived PrP^Sc^.

Immunohistochemical studies on the brainstem from cases confirmed as H-type from Germany [30] and the U.S. [16,31] showed that the prevalent PrP^Sc ^deposition pattern was less intense, intraneuronal and intraglial, unlike that of C-type BSE, which is more intense, with mainly granular and linear tract deposition patterns. At present, approximately 61 atypical BSE cases have been detected worldwide [as of March 2011].

In Italy, where both active and passive surveillance systems for BSE are in place, five out of 145 BSE cases detected to date were identified as BASE (L-type) variants. No H-type cases have been detected in Italy so far. However, the IHC and WB BSE confirmatory protocols in place in Italy (hereafter referred to as horse radish peroxidise [HRP] and whole homogenate extraction [WHE] methods) have proved successful in identifying PrP^Sc ^deposition in H-type BSE cases from Germany [30] and other European countries, respectively [21].

In non-European countries, and the U.S. in particular, current BSE surveillance programs focus on "high-risk cattle" defined as cattle displaying clinical symptoms consistent with BSE or fallen stock. U.S. surveillance has detected three BSE cases: one C-type and two H-type BSE have been identified [31]. The efficacy of the U.S. IHC and WB BSE confirmatory protocols (hereafter referred to as alkaline phosphatase [AP] and scrapie associated fibrils extraction [SAFE] methods, respectively) to identify L-type BSE forms has not yet been tested. The purpose of this study was to evaluate Italian and U.S. IHC and WB BSE confirmatory protocols in order to assess their suitability to detect C-, H- and L-type BSE cases. Tissue samples of classical and atypical BSE cases from Italy and the U.S., age-matched when possible, were tested by both the IHC or the WB methods used in each of the two countries.

## Results

### Immunohistochemical examination

#### Description of the PrP^Sc ^deposition patterns

Nine different PrP^Sc ^deposition patterns for the classical and atypical BSE cases were identified. The PrP^Sc ^deposition types, as described elsewhere [32,20], were:

**- punctate type **consisting of powdery and diffuse PrP^Sc ^staining throughout the neuropil;

**- granular type **characterized by granular PrP^Sc ^accumulations in the neuropil;

**- coalescing type **seemingly arising from the merging of granular PrP^Sc ^deposits to form amorphous or mesh-like masses;

**- glial type **with PrP^Sc ^deposits branching out from the nucleus of a glial cell on its processes, conferring it a stellate appearance;

**- intraneuronal type **with fine punctate PrP^Sc ^immunoreactivity throughout the neuronal cytoplasm;

**- perineuronal type **consisting in thread-like PrP^Sc ^deposits around individual neuronal perikarya and neurites;

**- intraglial type **with fine, punctate PrP^Sc ^immunoreactivity adjacent to the glial nuclei;

**- linear tract **characterized by PrP^Sc ^deposits along neuronal processes;

**- plaques **consisting of dense, generally rounded accumulations of PrP^Sc^, unicentric or occasionally multicentric, that may present a pale core surrounded by a dark border.

#### Comparative analysis of PrP^Sc ^deposition using AP and HRP methods

##### C-type BSE

**Figure 1:**
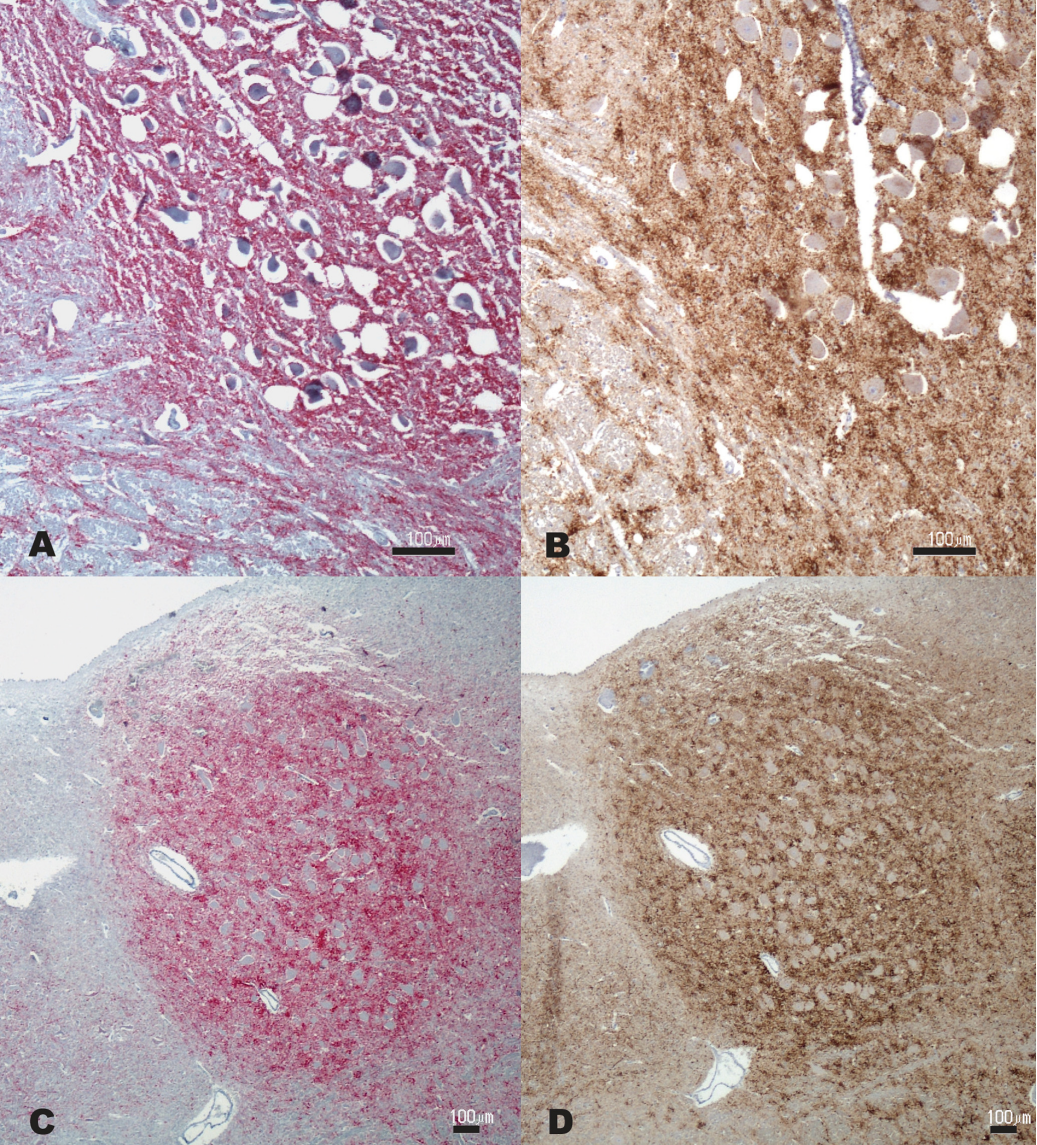
**C-type BSE**. (A and B) DMNV of the Italian case (16193/02); (C and D) NST of the U.S. case (B5330-7 D). (A and C) AP IHC method; (B and D) HRP IHC method. Bar = 100 μm.

The Italian C-type BSE (16193/02) obex tissue section stained by the AP method resulted in an overall marked intensity grade for PrP^Sc^. Moderate-to-marked punctate and granular, as well as intraneuronal and perineuronal type PrP^Sc ^deposition patterns were evident in the hypoglossal nucleus, dorsal motor nucleus of the vagus nerve (DMNV) (Figure 1A), nucleus of the solitary tract (NST), nucleus of the spinal tract of the trigeminal nerve (NSTV), reticular formation and olivary nucleus. Mild-to-marked coalescing and linear tract type PrP^Sc ^deposition was present in the reticular formation. In serial sections of the obex very similar results were obtained with the HRP method, with no detectable differences in PrP^Sc ^distribution, deposition pattern or staining intensity (Figure 1B).

The U.S. C-type BSE case (B5330-7 D) examined under the AP protocol contained overall moderate-to-marked PrP^Sc ^immunoreactivity. There was moderate punctate, granular, intraneural and perineuronal type PrP^Sc ^deposition in all the nuclei of the obex (Figure 1C). Glial, granular and linear tract PrP^Sc ^deposition patterns were mainly detected in the reticular formation.

The HRP protocol applied to serial sections of the U.S. C-type BSE case stained with the same neuroanatomical PrP^Sc ^distribution as seen under the AP protocol. Furthermore, PrP^Sc ^deposition patterns and related intensity for all the target nuclei appeared similar to the corresponding obex sections stained under the AP protocol (Figure 1D).

##### H-type BSE

**Figure 2:**
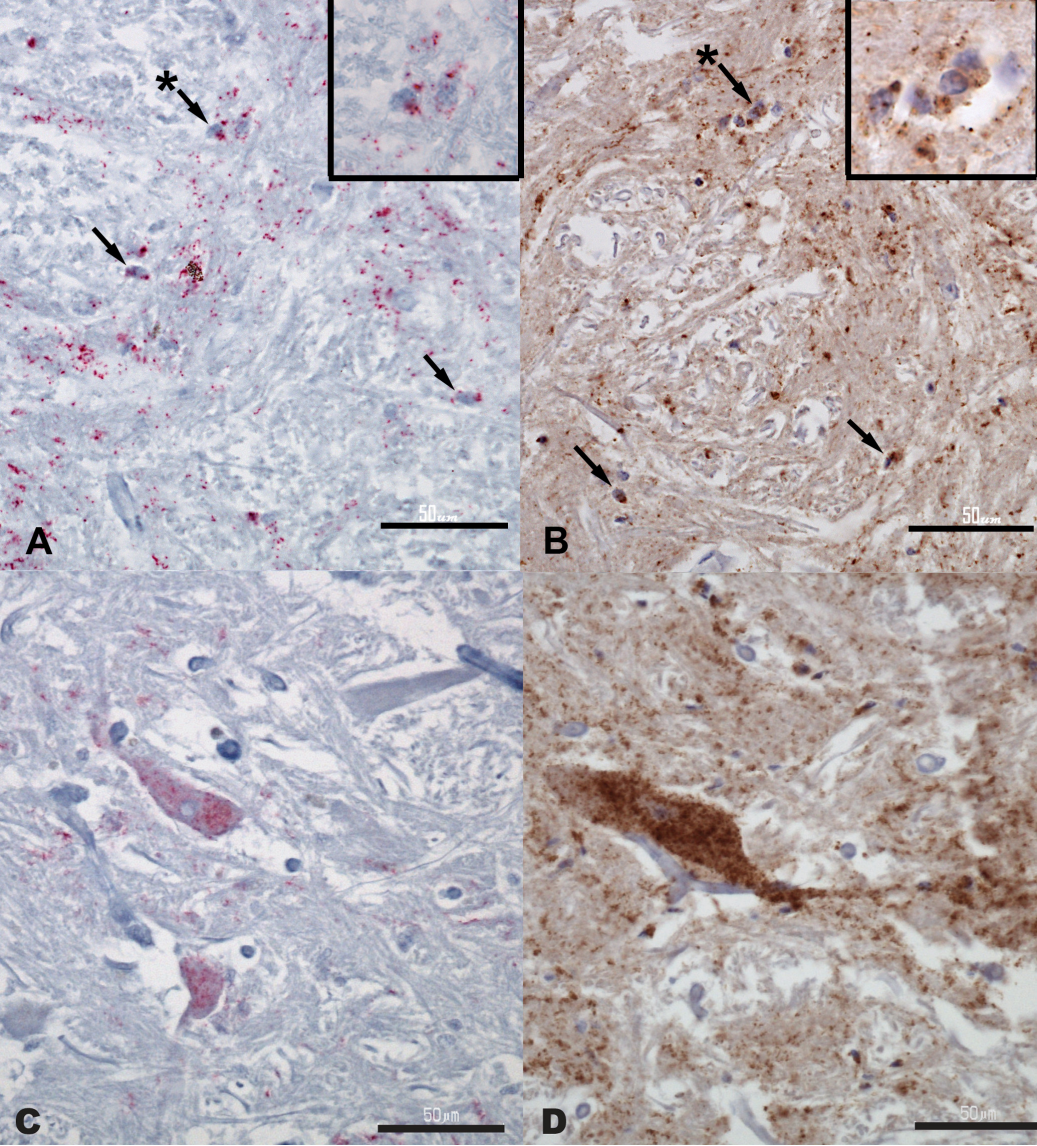
**U.S. H-type BSE (B14842): reticular formation**. (A and B) Intraglial PrP^Sc ^deposition pattern, marked by arrows; inset for each shows higher magnification for the marked glial cells indicated by the arrow with an asterisk. (C and D) Intraneuronal PrP^Sc ^deposition pattern. (A and C) AP IHC method; (B and D) HRP IHC method. Bar = 50 μm.

The H-type BSE case (B14842) showed overall moderate PrP^Sc ^immunoreactivity when stained under the AP protocol. PrP^Sc ^was mainly detected at the level of the DMNV, NST, NSTV and the reticular formation, primarily appearing as punctate and granular deposits with areas of intraglial deposition (Figure 2A). Intraneuronal and perineuronal PrP^Sc ^deposition was present in the DMNV and the reticular formation (Figure 2C).

Serial tissue sections of the same case stained under the HRP protocol revealed more widespread PrP^Sc ^distribution and moderate-to-marked immunolabelling intensity. PrP^Sc ^was detected through the whole tissue section. Interestingly, the HRP method revealed more intraglial (Figure 2B) and intraneuronal (Figure 2D) deposition patterns, with a higher degree of staining intensity than the AP method.

##### L-type BSE

**Figure 3:**
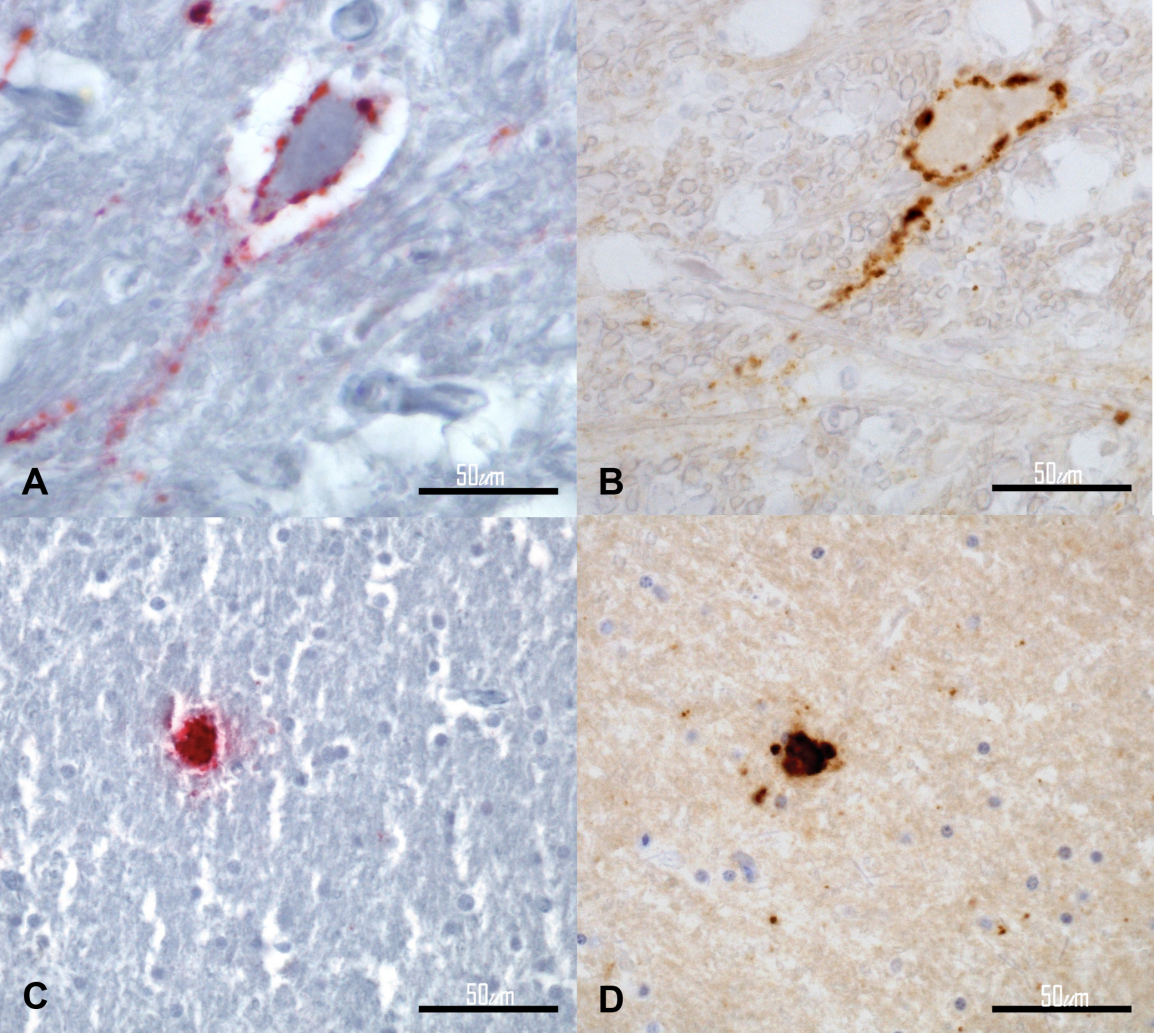
**Italian L-type BSE (BASE) (12966/07)**. (A and B) Perineuronal PrP^Sc ^deposition pattern of the DMNV; (C and D) Plaque-like PrP^Sc ^deposition pattern in the white matter of the frontal cortex. (A and C) AP IHC method; (B and D) HRP IHC method. Bar = 50 μm.

The L-type BSE (BASE) (12966/07) obex tissue sections examined under the AP protocol contained mild-to-moderate PrP^Sc ^immunoreactivity. The prevalent PrP^Sc ^deposition pattern was the punctate and granular type, which was mildly present in the hypoglossal and olivary nucleus and moderately present at the level of the DMNV, NST, NSTV, and reticular formation. Intraneuronal type PrP^Sc ^deposition was identified in some neurons of the hypoglossal nucleus, DMNV, NSTV, reticular formation and olivary nuclei. Perineuronal type PrP^Sc ^immunoreactivity of moderate intensity was identified in the DMNV (Figure 3A), reticular formation and the olivary nuclei. Linear tract type PrP^Sc ^deposits were present at the level of the reticular formation. Also present in the reticular formation were several larger aggregates of staining or plaques ranging from 5 to 25 μm in diameter. The majority of the plaques appeared as unicentric structures, but some were characterized by the presence of a pale core surrounded by a dark radial periphery.

The results obtained on the serial obex tissue sections of the BASE case stained according to the HRP method were comparable in terms of PrP^Sc ^distribution, staining intensity and deposition pattern (Figure 3B).

The frontal cortex of the BASE case stained under the AP protocol contained moderate punctate, granular type PrP^Sc ^deposition along the most superficial region of the cortex. In the deeper regions of the gray matter, a mild granular PrP^Sc ^deposition pattern was present. A similar pattern with moderate staining intensity was present along the transition zone between the gray and white matter. In the white matter there were granular PrP^Sc ^deposits and scattered PrP^Sc ^positive plaques that appeared as dense unicentric or multicentric, dark red, round structures (Figure 3C).

Serial tissue sections of the frontal cortex from this animal stained using the HRP procedure revealed a similar distribution of PrP^Sc ^deposits, including the PrP^Sc ^pattern types described above and comparable immunoreactivity intensity. The plaques appeared as dense, unicentric, dark brown, round structures, some of which displaying a pale core surrounded by a dark radial periphery (Figure 3D).

### Western blot analysis

**Figure 4:**
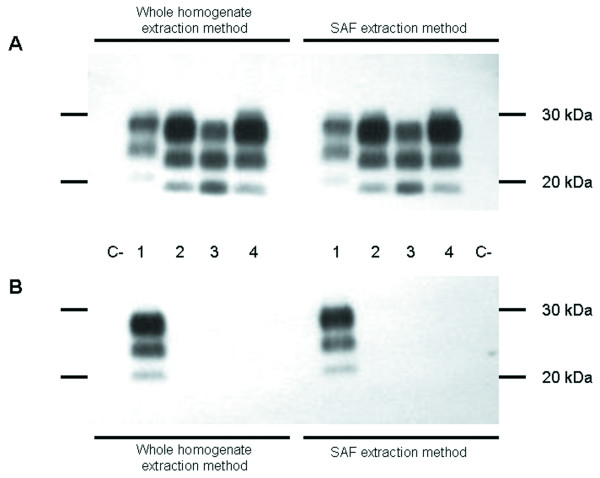
**Western blot analysis**. Healthy bovine used as negative control (C-), U.S. H-type BSE (1), Italian C-type BSE (2), Italian L-type BSE (3) and U.S. C-type BSE (4) samples, all examined by whole homogenate and SAF extraction methods. 10 milligrams of tissue equivalent were loaded per lane. All samples were proteinase K treated. Blots were probed with mAbs 6H4 (A) and P4 (B), respectively.

The obex tissue derived from a healthy bovine and used as control for PK digestion tested negative with both extraction methods. The WHE and SAFE extraction methods were both able to clearly identify classical and atypical BSE cases on subsequent WB analyses. When the 6H4 mAb was employed, which is directed against the protease-resistant core of the prion protein, all BSE samples (C-, H-, L-type BSE cases) tested positive (Figure 4A). In contrast, when the P4 mAb was used, which is directed against the N-terminus of the protease-resistant core of PrP, only the H-type BSE sample was recognized (Figure 4B) by both extraction methods. The density comparison between the signals obtained with P4 and 6H4 mAbs showed a P4/6H4 ratio of at least 1.2 for both methods in the H-type BSE sample. These findings were due to the different PK cleavage site for the three BSE types, as previously described by Jacobs et al. (21).

**Figure 5:**
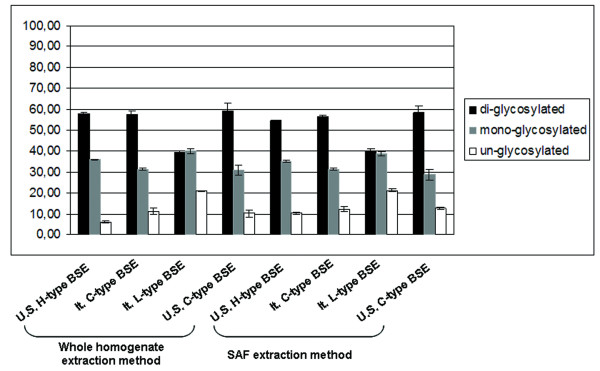
**Glycoform analysis**. Graphic representation of the proportions of the three PrP^Sc ^glycoforms of the samples examined by the whole homogenate and the SAF extraction methods.

In addition, the glycoform ratio of the different BSE samples was determined after staining with the 6H4 mAb. As shown in Figure 5, the L- and H-type BSE cases showed a lower proportion of the di-glycosylated and a higher proportion of the mono-glycosylated band than the C-type BSE cases according to both extraction methods. Specifically, the L-type BSE exhibited an equal or slightly inverted ratio between the mono- and the di-glycosylated PrP isoform, which is a characteristic feature of L-type BSE (Figure 5).

## Discussion and conclusions

The aim of this study was to evaluate the suitability of IHC and WB confirmatory tests routinely used in the U.S. and Italy for the diagnosis of classical and atypical BSE. In contrast to C-type BSE cases, which have progressively declined worldwide after the implementation of feed bans, atypical BSE (L- and H-type BSE) cases have been increasingly detected since 2004, and are postulated to arise as sporadic and genetic forms of the disease [14-16]. Most likely, the incidence of atypical BSE cases will be constant over time [14], as occurs in sporadic and genetic cases of human prion diseases [33]. The existence of atypical forms of BSE will require maintaining the current active surveillance systems for BSE; the misguided proposal many countries have put forward to progressively limit them could result in such cases being missed. Nonetheless, the feed bans on specified risk materials will continue to provide effective barriers for protecting public health.

All atypical BSE cases detected worldwide so far have been correctly identified by currently approved BSE rapid tests [34,35]. However, it should be noted that L-type BSE (BASE), the only BSE variant phenotypically characterized in detail to date [25], may contain significantly less PrP^Sc ^immunoreactivity in the obex region than C-type BSE. Since the obex is the target tissue for the diagnosis of all BSEs, it is necessary to evaluate the sensitivity of rapid testing and confirmatory diagnostic techniques currently in place for atypical forms. In the future, it may be necessary to re-evaluate whether the obex is the best tissue for detecting atypical BSE cases.

One aim of the present study was to assess the effectiveness of the U.S. BSE confirmatory methods to detect L-type BSE. The results indicate that the AP and SAFE BSE confirmatory protocols are fully adequate to identify the presence of PrP^Sc ^in the L-type atypical BSE forms. The efficacy of the AP and SAFE confirmatory methods to detect C- and H-type BSE has already been shown [16,31].

When the AP protocol was applied to obex and frontal cortex tissue sections of an Italian L-type BSE (BASE) case, PrP^Sc ^was detected with the same neuroanatomical distribution, deposition pattern and staining intensity as the HRP method. More specifically, the AP method revealed, at both the obex and frontal cortex, a characteristic neuropathologic trait of the Italian L-type BSE form, i.e., the presence of PrP^Sc ^aggregates in the form of plaques. Another peculiar feature described in cattle affected by natural [30] and experimental [36] L-type BSE is the presence of a perineuronal PrP^Sc ^deposition pattern which is not associated with intraneuronal PrP^Sc ^deposits. This PrP^Sc ^pattern was also detected by both the HRP and AP methods in the Italian L-type BSE case in this study. It has been hypothesized that the perineuronal PrP^Sc ^deposits could indicate different PrP^Sc ^trafficking and propagation in L-type BSE forms [36].

This comparative study also confirmed the reliability of the HRP and WHE confirmatory protocols in the diagnosis of the H-type BSE form not reported in Italy to date. Previous investigations performed on H-type samples obtained from other European countries revealed that the HRP technique was successful in detecting H-type BSE cases [30].

Under both the HRP and AP protocols, the U.S. H-type BSE case displayed a similar PrP^Sc ^deposition pattern of the granular, intraneuronal, and intraglial type. At present, only the obex and not a complete brain from an H-type BSE naturally affected cow has been available for analysis. In the obex region of H-type BSE cases, intraglial PrP^Sc ^deposition seems to be a peculiar immunohistochemical feature, as described in cases from Germany [30] and Sweden [37]. It is a less common feature for C- and L-type BSE cases [20,25]. Importantly, the intraglial pattern identified in a Swedish H-type BSE case was revealed only with antibodies (F99/97.6.1 and R145) directed against the C-terminus portion of the PrP^Sc ^molecule [37]. In contrast, the detection of severe granular intracellular labelling in German H-type BSE with the use of core-specific mAb 12F10 has also been reported [30].

The U.S. H-type BSE case examined here had more intense and defined PrP^Sc ^immunoreactivity of intraneuronal and intraglial distribution when tested using the HRP as compared to the AP method. This was surprising, since the AP protocol employs a ten-fold higher concentration of the primary F99/97.6.1 antibody as compared with the one employed by the HRP protocol. Moreover, as compared to the HRP method, the higher incubation temperature (37°C) at which the AP staining protocol occurs in the Ventana NexES carousel ought to confer a more efficient binding of the primary antibody to the tissue antigens. However, such preferable conditions in the AP method could partially be negatively influenced by a possible prozone-like effect due to the higher concentration of the primary antibody employed. The formation of antibody aggregates might limit their penetration into the cells and restrict binding to intracytoplasmatic antigenic sites [38]. Furthermore, it can not be excluded that the overall shorter incubation times of the AP protocol as compared to the HRP method might reduce the antibody-antigen interaction. These assumptions aside, the discrepancy between the AP and the HRP methods in the amount of PrP^Sc ^detected in the H-type case seems more likely to be related to the different length of the formic acid treatment performed on tissue sections after deparaffinization.

The HRP protocol utilizes a 25-min formic acid incubation, whereas the AP protocol limits formic acid exposure to 5 min. To perform immunohistochemical analysis on formalin-fixed tissues, a demasking step in formic acid is often used to expose antigenic sites to the binding by antibodies [39]; this step seems to be particularly critical to disclose the intracellular PrP^Sc ^component in H-type BSE cases. The PrP^Sc ^molecule characterizing H-type BSE probably requires prolonged exposure to formic acid to be properly demasked in formalin-fixed tissues and to be revealed, mainly by employing C-terminal antibodies, as intraneuronal and intraglial PrP^Sc ^deposits.

By contrat, a shorter demasking time in formic acid, i.e., 5 min as employed in the AP protocol, does not seem to affect the detection of the PrP^Sc ^plaque deposits, which may be a unique feature of L-type BSE cases. This finding suggests that in L-type BSE isolates the length of incubation time in formic acid is not a very critical step to disclose PrP^Sc ^deposits, perhaps indicating that L-type PrP^Sc ^most likely establishes weaker interactions with formalin and can therefore be more easily demasked than H-type PrP^Sc ^from formalin-fixed, paraffin-embedded tissue sections.

In summary, we found that comparative testing of an automated immunostaining protocol, with the use of the Ventana NexES Autostainer System, and a manual IHC method in the detection of PrP^Sc ^in various BSE cases revealed absolute concordance in correctly identifying BSE-positive samples and in discriminating among C-, H- and L-type forms of BSE. Both the HRP and the AP BSE confirmatory methods differentiated three different phenotypes of the disease, referred to as C-, H- and L-type forms, each appearing to be characterized by quite distinct features of PrP^Sc ^deposition. Both methods revealed granular and linear tract PrP^Sc ^deposits as a distinguishing feature of C-type BSE cases, whereas intraglial and intraneuronal PrP^Sc ^deposition patterns appeared as the most representative trait of H-type BSE, and the presence of PrP^Sc ^deposits organized as plaques was only detected in the L-type BSE (BASE) case.

In contrast to the IHC protocols, the WHE and the SAFE procedures for WB methods are almost identical. Both are, in fact, based on the enrichment of samples according to scrapie associated fibrils (SAF) immunoblotting methods derived from the World Organization for Animal Health (OIE) recommended protocol. The minor differences between the two protocols, for example, the use of different homogenization buffers or the sonication step, do not seem to affect results in terms of specificity and sensitivity to detect BSE cases. Furthermore, the fact that under both the WHE and SAFE protocols a higher amount of tissue equivalents (i.e., 10 mg) than that normally used for medium positive cases (i.e., ~ 500 μg) had to be loaded per lane is not to be ascribed to the sensitivity of the methods but simply to the need to display as best as possible the low signal intensity known to characterize the PrP^Sc ^moieties of some of the BSE cases under examination. Previous similar attempts to maximise at WB analysis the PrP^Sc ^signal intensity of slightly positive BSE samples by loading even higher amounts of tissue equivalents per lane (i.e., 38 mg) than the quantity employed here have also been reported (27).

Our findings show that both the SAFE and WHE methods were able to detect all three BSE forms (C-, H- and L-type). The WHE and SAFE methods identified and differentiated the specific molecular weights and glycoform ratios associated with the three BSE forms. As regards the H-type BSE in particular, it is noteworthy that both the SAFE and WHE methods clearly detected this atypical form, even when an antibody such as P4 was employed, which is known to be inferior for application to bovine PrP compared to other antibodies such as 12B2 (29) or 6H4. The results provide reliable evidence that the SAFE method can recognize and differentiate L-type BSE in the current surveillance program.

The present study proved that both Italy and the U.S. employ efficient diagnostic techniques to detect and differentiate the three different BSE forms (C-, H- and L-type) known to date. Furthermore, this finding suggests that the current Italian and U.S. confirmatory methods for BSE might also be appropriate to disclose novel forms of the disease should they ever occur.

## Methods

### U.S. tissues

The only C-type BSE case detected in the U.S. to date, identified as animal B5330-7 D, was examined. This animal was an approximately 6.5-year-old, as determined by dentition, non-ambulatory Holstein Friesian cow, imported from Canada in 2001 and slaughtered in Moses Lake, WA in 2003 [31] (Table 1). In accordance with the BSE surveillance system in place at that time, the case underwent confirmatory diagnosis by means of histology and IHC.

**Table 1:** Details of the C-, H- and L-type BSE cases examined

Case	Country	Age (year)	Breed	Cause of death	Husbandry	BSE type	Tissue	Tested in the study by
16193/02	Italy	5	Friesian	r.s.*	dairy	C-type	o.^#^	IHC
142585/03	Italy	11	Mixed	c.s.†	beef	C-type	o.^#^	WB
B5330-7 D	USA	~ 6.5	Friesian	c.s.†	dairy	C-type	o.^#^	IHC, WB
B10979	USA	~ 12	Brahma cross	f.s.‡	beef	H-type	o.^#^	WB
B14842	USA	~ 10	Red crossbred	f.s.‡	beef	H-type	o.^#^	IHC
1088/03	Italy	15	Piedmontese	f.s.‡	dairy/beef	L-type	o.^#^	WB
12966/07	Italy	14	Piedmontese	f.s.‡	dairy/beef	L-type	o.^#^, f.c.^§^	IHC

* r.s., routine slaughtered

† c.s., casualty slaughtered

‡ f.s., fallen stock

# o., obex

§ f.c., frontal cortex

Two H-type BSE cases, both U.S. born and identified as animals B10979 and B14842, were examined. Case B10979 was an approximately 12-year-old Brahman cross cow that died during transport to a packing plant [31] (Table 1). The case was first detected by rapid testing (Platelia/TeSeETM ELISA BSE test, Bio-Rad, Hercules, CA) and later confirmed positive by WB and IHC analysis in the U.S. and at the OIE Reference Laboratory for BSE in Weybridge, England. Case B14842 was an approximately 10-year-old crossbred (*Bos indicus × Bos taurus*) downer cow euthanized on the owner's farm by the herd veterinarian [16]. This case was also initially detected by rapid testing (Platelia/TeSeETM ELISA BSE test, Bio-Rad, Hercules, CA), then confirmed positive by IHC and WB analysis (Table 1).

### Italian tissues

Two C-type BSE cases were examined. Case 16193/02 was a 5-year-old routinely slaughtered Friesian cow and case 142585/03 was a slaughtered 11-year-old beef cow of mixed breed (Table 1). According to the Italian surveillance programs, both cases were first detected by a rapid screening test (Prionics Check Western, Prionics, Zurich, Switzerland) on the brainstem, and then confirmed by histology, IHC and WB analysis.

Two L-type BSE (BASE) cases, identified as animal 12966/07 and animal 1088/03 [25] were examined. Both cases were fallen stock Piedmontese cows, 14 and 15 years old respectively (Table 1), initially detected by rapid testing and then confirmed positive by BSE confirmatory tests.

### Immunohistochemistry (IHC)

#### Case selection and preparation of tissue sections

The Italian C-type BSE case (16193/02), U.S. C-type BSE case (B5330-7 D), Italian L-type (BASE) case (12966/07) and U.S. H-type case (B14842) were examined by IHC under both the HRP and the AP protocols. Obex sections for all cases and additional sections of the frontal cortex of the L-type case were tested (Table 1).

Serial sections were cut and numbered consecutively in order to test tissue sections, alternating between the AP protocol and the HRP method. Three slides of each tissue sample selected for the study were tested under the HRP protocol: two sections were incubated with the primary antibody; one, on which the primary antibody was omitted, was used as an internal control. Two slides of each tissue sample underwent IHC according to the AP protocol, and the primary antibody was applied to both of them.

The IHC staining comparison study was performed at the National Veterinary Services Laboratories (NVSL) in Ames, IA.

#### AP protocol

Tissues selected for examination were fixed in 10% buffered formalin, embedded in paraffin blocks, sectioned at 5 μm and mounted onto charged glass slides (Fisher Superfrost Plus, Fisher Scientific, Hampton, NH). The slides were air dried for a minimum of 3 h, put in an oven for 15 min at 80°C, deparaffinized in xylene, and rehydrated in graded alcohols, followed by distilled H_2_O. They were then incubated in 95% formic acid for 5 min, followed by three washes (2 min each) in Tris Buffer pH 7.5 and a 1-min wash in laboratory quality water. The slides were immersed in an antigen retrieval citrate buffer, pH 6 (DAKO™Target Retrieval Solution, Dako North America Inc., Carpinteria, CA) and placed in a commercially available antigen retrieval device (Biocare Medical, Concord, CA) for 30 min at 121°C. After the slides were allowed to cool to room temperature (RT), they were stained using an automated immunostainer (NexES IHC module, Ventana Medical Systems Inc., Tucson, AZ), previously filled with the PrP-specific F99/97.6.1 antibody (epitope QYQRES, aa 220-225 of the ovine PrP, 10 μg/ml dilution; VMRD Inc., Pullman, WA) [40], APK wash solution (Ventana Medical Systems Inc.) and liquid coverslip bottles (Dako North America Inc.). As suggested by the manufacturer, all incubations were performed on a Ventana NexES platform at 37°C following the alkaline phosphatase red paraffin protocols. The first step associated with the autostainer included incubation of sections with the F99/97.6.1 anti-PrP antibody for 32 min, rinsing and application of coverslip solution. The following steps were performed employing reagents provided by the Alkaline Phosphatase Basic Red Kit (Ventana Medical Systems Inc.) and involved incubation of the slides with a biotinylated goat antimouse IgG for 8 min, application of an avidin-alkaline phosphatase complex for 12 min, followed by incubation with an Enhancer reagent for a 4 min. Naphthol was used as substrate for the reaction and applied for 8 min together with Fast Red A, used as chromogen. Incubation with Fast Red B, used as second chromogen, followed for 8 min. The sections were subsequently counterstained with Gill's hematoxylin (Ventana Medical Systems Inc.) for 4 min and then post-counterstained with bluing reagent (Ventana Medical Systems Inc.) for 2 min. At the end of the process, the slides were removed from the automated stainer and dipped in warm tap water with 2-3 drops of liquid dishwashing detergent added (Non Ultra Dish Liquid, Original Scent, Dawn. Procter & Gamble, Cincinnati, OH). After washes in tap water, the sections were dehydrated in ethyl alcohol and xylene and then coverslipped with a permanent mounting medium.

Test slides were examined by light microscopy and classified as positive or negative for PrP^Sc ^immunoreactivity. PrP^Sc ^immunoreactivity was defined as pink to red staining; slides identified as negative for PrP^Sc ^contained only counterstaining which appeared blue. As positive controls, slides from the obex tissue of a scrapie positive sheep, obtained from the NVSL, and from the brainstem and the frontal cortex, respectively, of two experimentally infected TME positive cows, provided by the National Animal Disease Center (NADC), were employed. Obex tissue sections of an Italian BSE negative cow were utilized as negative controls.

#### HRP protocol

The Italian samples selected for the study were formalin-fixed and then treated for decontamination with 98% formic acid for 1 h before being embedded in paraffin blocks. Under the HRP protocol, sections were cut 5 μm thick for each sample, placed onto charged glass slides, drained, air dried for 10 min and then incubated at 37°C overnight. Dewaxing was performed in four 6-min steps: the first two in xylene, the next two in absolute ethyl alcohol. The slides were then rinsed and immersed in 98% formic acid for 25 min. After washing in distilled water, the rack of slides was put into a container filled with fresh epitope demasking solution (citrate buffer pH 6.1) with anti-bump glass beads at the bottom. The sections were then autoclaved for 30 min at 121°C. Endogenous peroxidase activity was blocked using 3% hydrogen peroxide in methanol for 20 min at RT. To block non-specific tissue antigens, the sections were incubated with 5% horse blocking serum for 20 min at RT, then incubated for 1 h at RT with the primary monoclonal antibody (MAb) F99/97.6.1 (epitope QYQRES, aa 220-225 of the ovine PrP, 1 μg/ml dilution; VMRD Inc.) [40]. After rinsing, a biotinylated goat anti-mouse secondary antibody (1:200 dilution; Vectastain ABC kit, Vector Laboratories, Burlingame, CA) was applied to the tissue sections for 30 min at RT, followed by the avidin-biotin-peroxidase complex (Vectastain ABC kit, Vector Laboratories) for a minimum of 30 min. PrP^Sc ^immunoreactivity was visualized using 3-3'- diaminobenzidine (Dakocytomation, Carpinteria, CA) as chromogen; the sections were then counterstained with Meyer's hematoxylin, dehydrated in ethyl alcohol and xylene and coverslipped. PrP^Sc ^immunoreactivity appeared from light to dark brown, whereas slides identified as negative for PrP^Sc ^staining contained only counterstaining which appeared blue. Positive and negative tissue controls for the HRP protocol were identical to the aforementioned controls used for the AP protocol.

#### Assessment of PrP^Sc ^immunoreactivity

For the C- and L-type BSE cases, the obex was sampled at the neuroanatomical level including the following nuclei: NST; DMNV; NSTV; the nucleus of the hypoglossal nerve; the olivary nucleus; the reticular formation. This allowed for the identification of PrP^Sc ^deposition and distribution.

For the L-type BSE (BASE) case, an area of frontal cortex was also examined.

Assessment of PrP^Sc ^immunoreactivity was based on neuroanatomical location, morphology and intensity (0, +, + +, + + +) where 0 denotes no PrP^Sc^, + denotes mild intensity, + + denotes moderate intensity, and + + + denotes marked intensity [20]. This assessment of PrP^Sc ^intensity is subjective.

### Western blot

Frozen obex samples from the Italian C-type BSE case (142585/03), U.S. C-type BSE case (B5330-7 D), Italian L-type (BASE) case (1088/03) and U.S. H-type case (B10979) and CNS tissue from a healthy bovine, used as negative control, were tested under both the WHE and the SAFE WB protocols (Table 1).

A portion of the Italian C- and L-type BSE cases was shipped to the Agricultural Research Service (ARS) Laboratories of the NADC, Ames, Iowa, where the WB comparison study was performed.

#### SAFE protocol

The OIE-recommended SAF-Immunoblot method [41] was used with minor modifications. Tissue homogenates (10% [w/v]) in 10 mM Tris, pH 7.5, containing 5 mM MgCl_2 _were prepared. The homogenates were sonicated for 30 s in an ice bath. Benzonase nuclease (100 U/ml; Novagen, Merck KGaA, Darmstadt, Germany) was added to the homogenates and they were incubated for 1 h at 37°C while shaking. An equal volume of 20% (w/v) sarcosyl in 10 mM Tris, pH 7.5 and 1 mM dithiotreithol (DTT) was added to each homogenate, vortexed for 1 min every 10 min for a total of 30 min at RT. The homogenates were transferred to polyallomer tubes and centrifuged at 20,000 *g *for 25 min at 10°C. Supernatants were collected and centrifuged again at 200,000 *g *for 55 min at 10°C. The resultant supernatants were discarded. The pellets were resuspended in sterile, distilled water (1 μl/mg tissue equivalent) and sonicated until suspended. The samples were treated with PK (40 μg/ml) by incubation at 37°C for 1 h with agitation. Phenylmethylsulphonyl fluoride (PMSF) was added to a final concentration of 5 mM, and the samples were incubated on ice for 15 min. The volume was brought up to 500 μl with water and centrifuged at 200,000 *g *for 1 h at 10°C. Supernatants were discarded and the pellets were resuspended in sodium dodecyl sulphate polyacrylamide gel electrophoresis (SDS-PAGE) sample buffer (Invitrogen, Carlsbad, CA).

#### WHE protocol

Samples were extracted by the WHE method previously reported [42]. Briefly, 10% (w/v) brainstem tissues were homogenized in sarcosyl-Tris buffered saline solution, pH 7.5. The homogenates were clarified by centrifugation at 22,000 *g *for 20 min at 10°C and the supernatants were incubated with PK (40 μg/ml) for 1 h at 37°C under continuous shaking. Digestion was stopped by adding 10 μl of PMSF 100 mM to the samples. After ultracentrifugation (Ultracentrifuge Optima TLX, Rotor TLA 110, Beckman Coulter, Fullerton, CA) at 215,000 *g *for 1 h at 10°C, the supernatants were discarded and pellets were resuspended in Laemmli buffer.

#### Western blot analysis

The samples extracted by the two methods were boiled for 10 min at 99°C, then separated by SDS-PAGE on 4-12% minigels (Invitrogen) and transferred onto polyvinylidene difluoride (PVDF) membranes (Immobilon-P, Millipore, Billerica, MA). Blots were blocked with PVDF blocking buffer (Invitrogen) for 1 h at RT, and blotted bovine PrP was detected with 6H4 (0.2 μg/ml; Prionics) [43] and P4 (0.1 μg/ml; R-Biopharm, Darmstadt, Germany) [28] monoclonal antibodies, respectively. After three washes with Tris buffered saline Tween 20 (TBST) (Invitrogen), the blots were incubated for 30 min at RT with a biotinylated sheep anti-mouse antibody (GE-Healthcare Ltd., St. Giles, UK). After another round of washes, streptavidin-horseradish peroxidase conjugate (GE-Healthcare Ltd.) incubation was done. Membranes were developed using ECL Plus chemiluminescent substrate (GE-Healthcare Ltd.) onto Hyperfilm ECL (GE-Healthcare Ltd.). The photographic films were then recorded using the UVI-prochemi gel documentation system (Uvitec, Cambridge, UK).

#### P4/6H4 mAbs ratio evaluation and glycoform analysis

The ratio between P4 and 6H4 mAbs was calculated by examining the films at equal time exposure, taking into account only the H-type sample signals. Samples were run three times and the films were analyzed using UVI-prochemi software (Uvitec).

A glycoform analysis was performed to better determine whether the glycoform pattern of the three PrP^Sc ^of the different BSE strains was detected by both extraction protocols. The samples were examined in three runs to evaluate the variability of the methods. Signals detected with mAb 6H4 were examined using UVI-prochemi software (Uvitec) and the values obtained for the di-glycosylated, mono-glycosylated and un-glycosylated bands were plotted as a graph.

## Competing interests

The authors declare that they have no competing interests.

## Authors' contributions

JAR, MC, CC^# ^and PA conceived of the study. JAR, SMH, CC^¥^, CC^#^, FM, CP and ANH participated in its design. JAR, SMH, CC^¥^, FM, CP, AZP coordinated the study. ANH kindly provided the TME infected cattle tissue as positive control. CP and CML performed the immunohistochemical investigations, FM and JAR carried out the Western blot analyses. JAR and CC^# ^helped to draft the manuscript. CP and FM wrote the manuscript. CML, SMH, JJG, JAR, CC^#^, CC^¥^, BI, PA and FM contributed to manuscript revision. All authors have read and approved the final manuscript.
